# Terrestrosin D from *Tribulus terrestris* attenuates bleomycin-induced inflammation and suppresses fibrotic changes in the lungs of mice

**DOI:** 10.1080/13880209.2019.1672754

**Published:** 2019-10-14

**Authors:** Min Qiu, Ming An, Mengni Bian, Shunbang Yu, Changxiao Liu, Quanli Liu

**Affiliations:** a Department of Pharmacy, Baotou Medical College, Baotou, China;; b School of Health Sciences, University of Newcastle, Newcastle, Australia;; c Tianjin Institute of Pharmaceutical Research, Tianjin, China

**Keywords:** Anti-fibrosis pharmaceutical, anti-inflammatory agent, bleomycin-induced pulmonary inflammation, pulmonary fibrosis, hydroxyproline

## Abstract

**Context:** Terrestrosin D (TED), from *Tribulus terrestris* L. (Zygophyllaceae), exhibits anti-tumour and anti-inflammatory activities. However, its effects on bleomycin (BLM)-induced pulmonary inflammation and the subsequent fibrotic changes remain unclear.

**Objective:** To examine the anti-inflammatory and anti-fibrotic effects of TED against BLM in murine pulmonary tissues.

**Materials and methods:** Male SPF mice received saline (control), TED (10 mg/kg), BLM (2.5 mg/kg), or BLM (2.5 mg/kg) + TED (10 mg/kg) group. BLM was administered as a single intranasal inoculation, and TED was intraperitoneally administered once daily. After 2 and 6 weeks of treatment, cell number and differentiation (Giemsa staining) and TNF-α, IL-6, IL-8, TGF-β1, and PDGF-AB levels (ELISA) were determined in the bronchoalveolar lavage fluid (BALF). Hydroxyproline (Hyp) content in the left pulmonary tissue was also determined (ELISA). The right pulmonary tissue was H&E-stained and assessed for the severity of pulmonary fibrosis using the Ashcroft scoring method. Compared with the BLM group, TED decreased inflammatory cell infiltration; number of macrophages (*p* < 0.05), neutrophils (*p* < 0.05), lymphocytes (*p* < 0.05); percentage of macrophages in the monocyte-macrophage system (*p* < 0.05), and levels of TNF-α (*p* < 0.01), IL-6 (*p* < 0.01), IL-8 (*p* < 0.05), TGF-β1 (*p* < 0.05), and PDGF-AB (*p* < 0.05) in the BALF. TED also reduced Hyp content (*p* < 0.05) in the pulmonary tissue and attenuated the BLM-induced deterioration in lung histopathology.

**Discussion and conclusions:** TED can inhibit BLM-induced inflammation and fibrosis in the lungs of mice, which may be related to reduced inflammatory and fibrotic markers. These results could be further tested in humans through clinical studies.

## Introduction

Pulmonary fibrosis is a cryptogenic diffusive pulmonary disease, which is the end-stage change of a wide range of pulmonary diseases (Geiser 2003; Choi et al. 2018). Pulmonary fibrosis consists of two stages with distinct pathological features: the early inflammatory stage, where alveolar inflammatory injuries result in damage to the tissue structure, and the fibrotic end stage, where disordered repair of inflammatory injuries produce aberrant tissue structures. Pulmonary fibrosis can greatly affect respiratory function, resulting in dry cough and progressive dyspnea, where the respiratory function of the patient may deteriorate continuously with the progression of the pathology, resulting in respiratory failure. Currently, the morbidity and mortality of pulmonary fibrosis is increasing annually due to a lack of effective pharmaceutical interventions. The average survival time following diagnosis is 2–4 years (Aravena et al. 2015; Spagnolo et al. 2015), corresponding to a death rate higher than most kinds of tumours and accounting for its designation as a ‘tumour-like disease’ (Li et al. 2019). In USA, the FDA has listed two drugs as treatment options: pirfenidone (Esbriet^®^) and nintedanib (Ofev^®^). However, these drugs have been known to cause adverse effects including rash, diarrhoea, nausea, and hepatic dysfunctions (Rogliani et al. 2016; Ren et al. 2017). Therefore, new drugs need to be developed and implemented in the clinical treatment of the disease to improve the post-diagnosis living qualities of patients and improve the prognosis of endpoints.

Bleomycin (BLM) is a type of glycopeptide antibiotic with multiple anti-tumour effects initially isolated from the bacterium *Streptomyces verticillus* (Duecker et al. 2018). Previous studies have indicated that the oxidative stress induced by BLM participates in the occurrence of pulmonary fibrosis. The pathological process after the administration of BLM was highly similar to that of human idiopathic pulmonary fibrosis (Sun et al. 2018). After intra-tracheal instillation or intranasal inoculation, the BLM-Fe^2+^ compound is formed, which produces reactive oxygen species that damage DNA, leading to inflammatory responses in the early stage, fibrotic changes in the alveolar mesenchyme and extensive proliferation of fibroblasts during the later stage, and the formation of fibrosis in the final stage.

The dried mature fruit of the annual herb *Tribulus terrestris* L. (Zygophyllaceae) (Pavin et al. 2018) is a commonly used traditional Chinese-Mongolian medicine. Its main effective constituent is terrestrosin, which is primarily composed of steroid saponins (Yan et al. 1996; Bedir et al. 2002; Neychev et al. 2007). Modern pharmacological research has demonstrated the effects of steroid saponins against tumours, cardiovascular and cerebrovascular diseases, senescence, and inflammation (Gauthaman et al. 2002; Ghosian Moghaddam et al. 2013; Sharifi et al. 2014). The significant anti-inflammatory effects of steroid saponins originate from the *N-trans*-ρ-caffeoyl tyramine structure (Kang et al. 2017). As demonstrated by Kang et al. (2017), terrestrosin suppresses oxazolone-induced dermatitis in mice and has significant anti-inflammatory effects in lipopolysaccharide-induced mouse inflammation models. Wei et al. (2014) also demonstrated that terrestrosin D (TED) was the configuration with pharmacological functions. Currently, the effects of TED against pulmonary inflammation and the subsequent fibrotic changes are unknown.

In this study, we observed the effects of TED against BLM-induced pulmonary inflammation and fibrosis in the lungs of mice by quantifying the changes in cell populations and differentiation, inflammatory factor expression levels, and structural and cellular morphology in the lungs of mice.

## Materials and methods

### Animals and ethics statement

SPF grade male KM mice (80 total), were bought from SPF Biotechnology Co. Ltd. (Beijing) batch code SCXK (Beijing) 2016-0002. Upon arrival, the mice weighed 30 ± 2 g. Before treatment, they were acclimatized for 1 week, and maintained at room temperature (22 ± 3 °C), with a humidity of 45 ± 5%. The Ethics Committee of Baotou Medical College approved use of animals in this study.

### Chemicals

**Figure 1.**
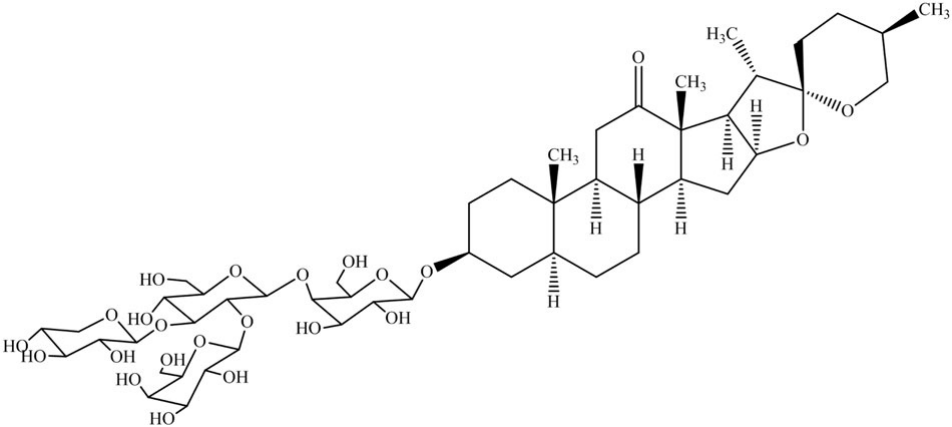
Structural formula of TED.

TED was purchased from the National Institutes for Food and Drug Control (the chemical structure is shown in Figure 1): batch number 112022-201601, CAS number 179464-23-4, and purity 95.3%. Before administration, TED was suspended in saline at a concentration of 2 mg/mL. BLM was purchased from Abcam Biotechnology (Cambridge, MA, USA, Catalog No. ab142977). Before administration, BLM was solubilized in saline at a concentration of 1.5 mg/mL.

### Animal treatment

The 80 SPF grade male KM mice were randomly divided into four groups (*n* = 20), each treated with different strategies: the physiological saline control group, the TED administration group (10 mg/kg body weight), the BLM administration group (2.5 mg/kg body weight), and the BLM/TED co-administration group (BLM 2.5 mg/kg body weight – TED 10 mg/kg body weight). Before administration, the mice were anesthetized with 1% pentobarbital sodium via intraperitoneal injection at a dose of 10 mL/kg body weight. All groups were administered physiological saline or TED intraperitoneally (IP) following intranasal administration of BLM for either 2 weeks or 6 weeks continuously. Ten mice from each group were randomly chosen and sacrificed for sample collection at the 2-week time point. The remaining mice were sacrificed for sample collection at 6 weeks.

### Sample collection

After reaching the time-point for sample collection, the mice were anesthetized in accordance with the above protocols. Followed by anaesthesia, cardiac perfusion was performed to obtain a blood-free pulmonary sample. After perfusion, the left lung was ligated. Bronchial-alveolar lavage was performed with saline (2 mL in, and the volume of the retrieved fluid was recorded as *V_BALF_*). The obtained fluids were centrifuged under 3000 rpm for 10 min, and the supernatant was stored under −80 °C in order to analyze the selected biomarkers by ELISA. By using the Giemsa staining method, the sediment cells at the bottom of the tubes were resuspended and centrifuged in PBS for differentiating cell populations. The perfused right lung was retrieved and stored in fixate (10% formaldehyde) for Histopathological examination, stained by adopting the H&E method afterwards. The ligated left lung to be detected by ELISA was retrieved and stored under −80 °C for hydroxyproline (Hyp).

### Giemsa staining (cell populations and differentiation in BALF)

The cell suspension in PBS obtained from BALF was centrifuged under 800 rpm onto glass slides with Cytospin (Thermofisher, USA). Giemsa Staining (Sigma-Aldrich, Lot # SLBT6353) was performed according to the manufacturer’s instructions. The obtained slides were examined under the optical microscope (Olympus, Japan) to analyze the cell population quantification. Based on the cell counts per microscopic sight per unit volume of BALF (defined here as *C*), the cell density was calculated. Specifically, five recordings per sample were made for the number of the cells per sight (*n*), and *C* was calculated via the following formula:

$$C=\frac{n}{V_{\mathrm{BALF}}}$$

When analyzing the monocyte/macrophage quantification, the data were acquired by counting 400 cells per slide. As for the neutrophil/macrophage/lymphocyte quantification analysis, the data were acquired by counting 500 cells per slide.

### ELISAs (inflammation and fibrosis quantification in BALF)

ELISA kits were used to determine the activity of tumour necrosis factor α (TNF-α) (R&D, Catalog No. MTA00B), interleukin-6 (IL-6) (R&D, Catalog No. M6000B), interleukin-8 (IL-8) (MYBioSource, Catalog # MBS261967), transformation-growth factor β1 (TGF-β1) (R&D, Catalog No. MB100B), and PDGF-AB (R&D, Catalog No. MHD00) coming from the BALF supernatant followed by the manufacturer’s instructions. The ligated left lung was analyzed for hydroxyproline (Hyp) quantification by using the Hyp ELISA kit (MYBioSource, Catalog # MBS703512). Assays were performed in duplicate wells, and the absorbance at 450 nm was measured with a microplate reader (Thermo Scientific^TM^).

### Histopathological examination

The fixed right lung sample was embedded in paraffin, and was cut into 5-μm sections. The obtained slides were stained with the standard H&E staining method, and examined under the optical microscope. The standardized morphological criteria were used to determine the relevant cellular morphology. Each successive field was individually assessed for severity of tissue fibrosis and allotted a score between 0 and 8 using a predetermined scale of severity (Ashcroft et al. 1988):

0: Normal pulmonary tissue.
1: Minimum thicknesses of alveoli and bronchiole walls.
2–3: Medium thicknesses of alveoli and bronchiole walls without significant damage to lung structure.
4–5: Increase in fibrosis with significant damage to lung structure and formation of small clumps of fibrotic tissue.
6–7: Severe destruction of lung structure, large areas of fibrosis and ‘honeycomb lung’.
8: Total fibrosis of lung.

### Statistical analysis

All data presented as mean ± SEM were subjected to one-way analysis of variance (ANOVA) using SPSS 19.0 software (SPSS Inc., Chicago, IL, USA) and Fisher’s Least Square Difference (LSD) test employed to determine significance. The criterion for significance was set at *p* < 0.05.

## Results

### Cell populations and differentiation in BALF

**Figure 2.**
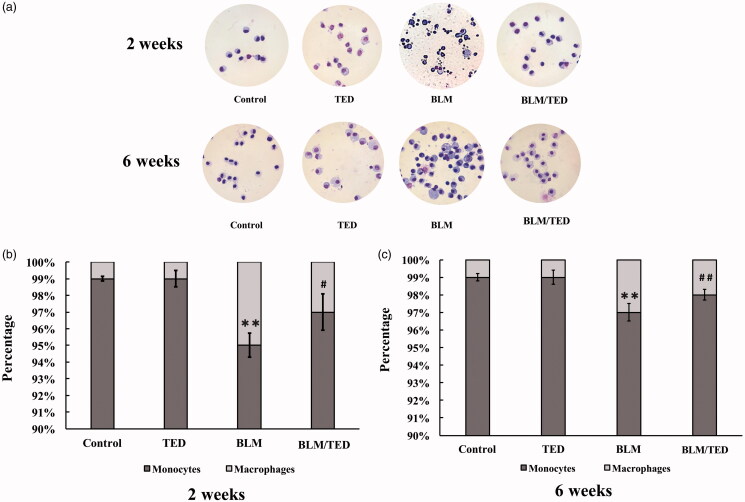
Images of cells in BALF and the percentages of monocytes and macrophages in the monocyte-macrophage system from different groups of mice at different time points. A shows the images of BALF cells from different groups of mice by 2 and 6 weeks treated with Giemsa staining (10 × 100). C: Cell counts; M: Macrophages; N: neutrophils; L: lymphocytes. B and C show the percentages of monocytes and macrophages by 2 and 6 weeks, respectively. Data presented are mean ± SEM of 10 mice per group. **p < 0.01 *vs* Control. ^#^*p* < 0.05 and ^##^*p* < 0.01 *vs* BLM.

BLM induces severe inflammatory responses. Following BLM treatment, significant changes were observed in the BALF (Figure 2(A)). Specifically, reflected by *C*, the cell density rose by approximately 4-fold in week 2, indicating severe inflammatory responses in the bronchial-alveolar cavities (Table 1). In the monocyte-macrophage system, the percentage of monocytes declined, while the percentage of macrophages increased by approximately 5-fold during the early period of inflammation, indicating chemotaxis of monocytes and activation of macrophages (Figure 2(B)). This change persisted into the later period of inflammation (6 weeks), where the percentage of macrophages dropped slightly, suggesting that the inflammation had ‘eased’ into the chronic phase (Figure 2(C)). Among the leukocytes, the proportion of neutrophils and lymphocytes rose significantly during the 2-week period, suggesting strong chemotactic reactions (Table 1). After 6 weeks, *C* only exhibited a negligible decrease, indicating that the inflammation had turned chronic (Table 1). Simultaneously, the percentage of neutrophils exhibited no change, and the percentage of macrophages and lymphocytes increased and declined, respectively, indicating an elevation in the macrophage population and a drop in the lymphocyte population. This suggests that the inflammatory responses ‘ebbed’ (Table 1).

**Table 1.** Cell populations and differentiation in BALF from different groups of mice at different time points.

Groups	2W				6W			
	C/(sight·ml)	M (%)	N (%)	L (%)	C/(sight·ml)	M (%)	N (%)	L (%)
Control	3.93 ± 0.63	98.80 ± 1.00	0.20 ± 0.07	1.00 ± 0.08	3.94 ± 0.67	98.96 ± 0.04	0.36 ± 0.01	0.68 ± 0.20
TED	3.81 ± 0.51	98.90 ± 1.00	0.10 ± 0.03	1.00 ± 0.06	4.00 ± 0.62	99.00 ± 1.30	0.00 ± 0.00	1.00 ± 0.20
BLM	18.10 ± 2.68**	94.48 ± 1.00**	2.12 ± 0.40**	3.40 ± 0.30**	17.26 ± 1.89**	95.28 ± 1.50**	2.04 ± 0.03**	2.68 ± 0.40**
BLM-TED	9.03 ± 1.02^##^	96.80 ± 1.00^##^	0.20 ± 0.02^##^	3.00 ± 0.04^##^	10.80 ± 1.30^##^	97.36 ± 0.40^#^	1.36 ± 0.03^#^	1.28 ± 0.20^#^

Data presented are mean ± SEM (*n* = 10).

**p < 0.01 *vs* Control.

^#^*p* < 0.05 and ^##^*p* < 0.01 *vs* BLM.

TED suppresses the BLM-induced inflammatory responses. From the Giemsa staining, we observed an alleviation of the BLM-induced inflammatory responses by TED (Figure 2(A)). In comparison to the BLM group, *C* demonstrates that the cell density in the BLM/TED group is approximately half of that in the BLM group (Table 1), indicating significant suppression of inflammation. In the monocyte-macrophage system, the macrophage percentage declined, suggesting suppression of macrophage chemotaxis and activation (Figure 2(B)). In the leukocyte population, the percentages of macrophages, neutrophils, and lymphocytes returned to levels comparable to that in the control group by week 6 (Table 1), indicating amelioration of inflammatory responses.

### Inflammation and fibrosis

**Figure 3.**
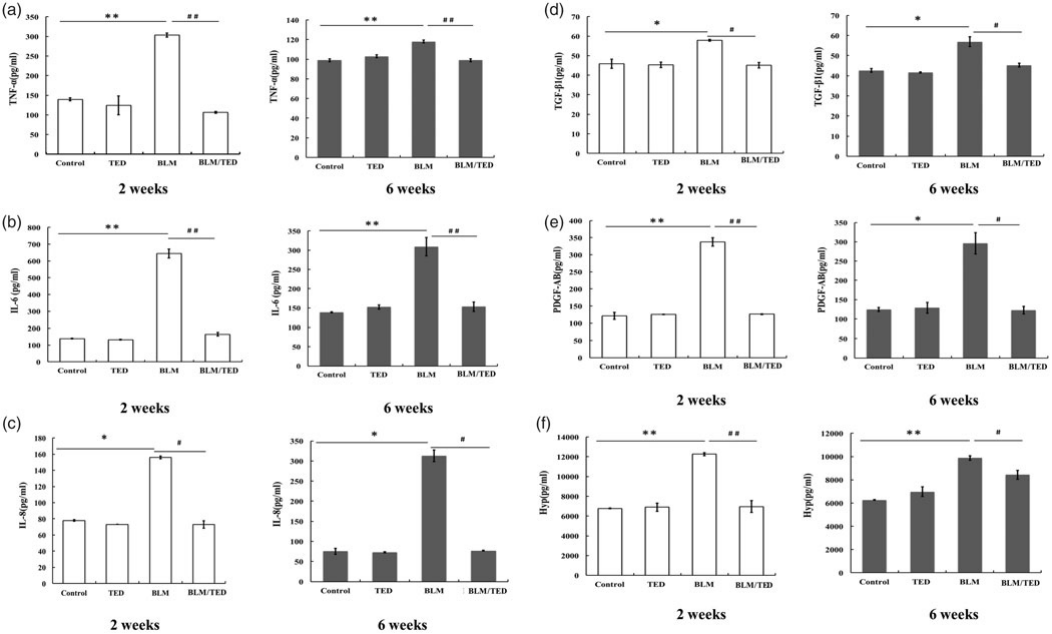
Quantification of inflammatory and fibrotic factors in different groups of mice at different time points. (A–C) ELISA for TNF-α, IL-6 and IL-8 quantification, respectively, in BALF collected 2 and 6 weeks after intranasal administration. (D–E) ELISA for TGF-β, PDGF-AB quantification, respectively, in BALF collected 2 and 6 weeks after intranasal inoculation administration. (F) ELISA for Hyp assay to quantify collagen expression in the lungs of mice 2 and 6 weeks after intranasal administration. Data presented are mean ± SEM (*n* = 10). *p < 0.05 and **p < 0.01 *vs* Control. ^#^*p* < 0.05 and ^##^*p* < 0.01 *vs* BLM.

BLM initiates inflammatory responses and fibrotic changes. Following treatment with BLM, intensive inflammation was observed in the BALF, with TNF-α, IL-6, IL-8, and TGF-β1 being upregulated during the early period of inflammation (2 weeks) (Figure 3(A–C)). During the late period of inflammation and initiation period of fibrosis (6 weeks), the expression levels of TNF-α and IL-6 decreased, while the expression level of IL-8 doubled, indicating a continuous and progressive inflammatory response. The expression levels of TGF-β1, PDGF-AB, and Hyp exhibited increases of approximately 1.5-, 3- and 2-fold, respectively, as a result of BLM treatment, and showed no significant changes from 2 weeks to 6 weeks, indicating fibrotic accumulation during the entire process (Figure 3(D–F)).

TED inhibits inflammation and fibrotic accumulation. Introduction of TED significantly reduced the damaging process initiated by BLM. Specifically, TNF-α, IL-6, and IL-8 were reduced to levels similar to the control group, which provides evidence that TED inhibited fibrosis from an early stage by containing the inflammation. TGF-β1, PDGF-AB, and Hyp expression levels were also suppressed to levels comparable to those in the control group, suggesting that the final fibrotic stage was inhibited (or at least delayed) by the introduction of TED (Figure 3).

### Histopathology

**Figure 4.**
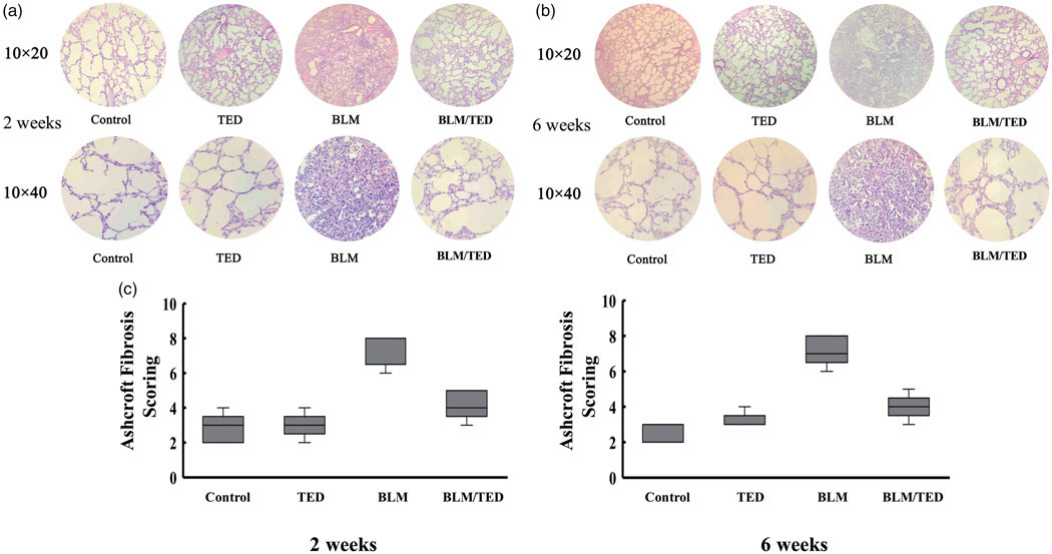
Immunohistochemical images of the pulmonary tissues from different groups of mice at different time points and quantification of fibrotic changes via modified Ashcroft Scoring System. A and B show the low (10 × 20) and high magnification (10 × 40) images of the tissues to demonstrate the changes in tissue structure and cellular morphology by 2 and 6 weeks, respectively. 4C shows the results of the modified Ashcroft Scoring method. Data presented are mean ± SEM (*n* = 5). *p < 0.05 and **p < 0.01 *vs* Control. ^#^*p* < 0.05 and ^##^*p* < 0.01 *vs* BLM.

BLM induces fibrotic changes in the pulmonary tissue. BLM treatment for 2 weeks decreased the alveolar space (Figure 4(A)), whereas treatment for 6 weeks resulted in the formation of large focal areas due to the accumulation of fibroblasts in the pulmonary tissue (Figure 4(B)). Microscopy analysis revealed the accumulation of elongated cells, suggesting fibroblast recruitment to the pulmonary tissue (Figure 4(A,B). Average Ashcroft scores were 7.4 (2 weeks) and 7.2 (6 weeks) after the single intranasal inoculation of BLM (Figure 4(C)).

TED intervention inhibits fibrotic changes. The application of TED greatly alleviated the reduction observed in the alveolar space, and the fibrotic changes were significantly suppressed compared to those observed in the BLM group (Figure 4). A slight increase in the alveolar wall thickness was observed, though the overall quality of the pulmonary structure was significantly improved. Under high magnification, we observed no significant changes among the cell populations. The Ashcroft Score (modified version) of the BLM/TED group consistently decreased to an average score of 4.2 (2 weeks) and 4 (6 weeks) in comparison to those of the BLM group (Figure 4(C)).

### TED has no adverse effects in terms of inflammation or fibrosis

TED alone has no effect on leukocyte differentiation and selected biomarkers. In the TED group, the results of cell differentiation analysis indicated no changes, suggesting that TED alone induces no effects on the leukocytes (Figure 2). Comparison of expression levels for TNF-α, IL-6, IL-8, and TGF-β1 between the control groups and TED-treated groups (no BLM, 2 weeks and 6 weeks of treatment) showed no significant statistical difference, indicating that TED alone had no negative effects on the pulmonary tissues (Figure 3). Additionally, no significant differences were observed for measurements of TGF-β1, PDGF-AB, and Hyp expression levels between the control and TED-treated groups.

TED alone has no effects on histopathology. No changes were observed through histopathological examinations of the TED group following administration. Specifically, histomorphological images demonstrate no structural changes in the lungs. Under high magnification, no morphological changes were observed among the tissue cells (Figure 4). The results were consistent with the Ashcroft (modified) Scoring System, which produced an average score of 3 (2 and 6 weeks) in the TED group.

## Discussion

In the present study, we examined the effects of TED in BLM-induced mouse models of pulmonary fibrosis. We provided evidence sufficient to demonstrate that TED effectively suppressed the inflammatory responses and fibrotic changes observed following exposure to BLM. From the results obtained, we conclude that TED inhibited BLM-induced inflammation from the initiation period and no inflammation-independent fibrotic changes occurred.

Previous research papers on inflammation and fibrosis have discussed the roles of neutrophils, macrophages, and lymphocytes, among which macrophages have been emphasized for their major role in the development of inflammation. In human lungs, macrophages are the predominant resident cells in the alveolar spaces (Wigenstam et al. 2016). During the initial period of inflammation, inflammatory cells (including macrophages) secrete IL-8, which is a chemokine that recruits neutrophils and monocytes to the site of inflammation (Rajagopal et al. 2015). Under the inflammatory microenvironment, the recruited neutrophils and monocytes are activated to secrete cytokines, including TNF-α, IL-6, IL-8, and TGF-β1 (Choi et al. 2018). Additionally, inflammatory injuries may result in TGF-β1 and PDGF-AB secretion by vascular endothelial cells and platelets (Nishioka et al. 2013; Rajagopal et al. 2015). During the later period of inflammation and initiation period of fibrosis, TGF-β1 and PDGF-AB play stimulative roles. As a chemotactic agent, TGF-β1 induces the chemotaxis of macrophages, the epithelial-mesenchymal transition (EMT, which contributes to the lung fibroblast population), thereby of collagen and synthesis of the extracellular matrix, which results in fibrosis (Zhang et al. 2018). Similar to TGF-β1, PDGF-AB is a chemotactic agent and a potential mitogen that induces the chemotaxis and proliferation of monocytes, macrophages, and mesenchymal fibroblasts, thereby consolidating inflammation and promoting fibrosis by accelerating formation of the extracellular matrix and collagen (Pierce et al. 1991; Lindroos et al. 1997; Tuleta et al. 2018). As the main component of collagen, Hyp accounts for approximately 13% of the total amino acids and is an important targeted indicator of collagen metabolism (Sun et al. 2018). Therefore, along with TGF-β1 and PDGF-AB, Hyp has been utilized as a biomarker of fibrosis.

In classic models of pulmonary fibrosis established with BLM, induction of inflammation was known to occur on the 3rd day following BLM administration. The inflammatory responses peak during the second week before transforming to chronic inflammation after 2 weeks, followed by the initiation of fibrotic changes. Fibrosis is usually established between 4 and 6 weeks (Kim et al. 2018). In this research, we quantified the effects of TED against BLM-induced pulmonary inflammation and fibrosis by quantifying inflammatory responses and fibrotic changes. Specifically, biomarkers were recruited to distinguish the two different stages in the inflammation-fibrosis pathway: the initiation of inflammation (TNF-α, IL-6, and IL-8) and the accumulation of fibrotic changes (TGF-β1, PDGF-AB, and Hyp).

The BALF analysis quantified the changes in the cell populations of the four groups. The total cell count in the BLM group increased compared to that in the control group. Simultaneously, the percentage of neutrophils and lymphocytes rose, consistent with the characteristics of inflammation (Fisichella et al. 2013). Additionally, the percentage of monocytes in the monocyte-macrophage system declined, indicating increased chemotaxis of monocytes. In contrast, the percentage of macrophages, neutrophils, and lymphocytes in the BLM/TED group remained comparable to that of the control group, suggesting that the inflammatory response driven by administration of BLM was alleviated. Additionally, the monocyte population in the monocyte-macrophage system of the BLM/TED group was revived.

Consistent with the classic BLM model and cell differentiation analysis results, BLM treatment in our study resulted in an increase in IL-8 expression levels during the initial 2-week period, which continued to rise to approximately twice the 2-week level by 6 weeks, indicating a continuous and progressive inflammatory response among the alveolar macrophages. Additionally, the elevated expression levels of TNF- α and IL-6 in the BLM group indicated activation of newly recruited macrophages in the pulmonary tissues in response to the inflammation. The TGF-β1, PDGF-AB, and Hyp levels were increased in the BLM group compared to that in the control group, indicating a progressive fibrotic accumulation simultaneous with the initiation and progression of inflammation. Following treatment with TED, we observed that the expression levels of the inflammatory markers dropped, suggesting an alleviation of inflammation. It should be emphasized that like TNF-α and IL-6, the expression level of IL-8 dropped to a level that was somewhat comparable to that in the control group, which suggests that the inflammatory cells that initiated the BLM-induced inflammation were ‘pacified’. From the expression levels of TGF-β1, PDGF-AB, and Hyp in the BLM/TED group, we infer that the fibrotic accumulation was delayed by administration of TED. The reduced expression level of TGF-β1 suggests to us that the initiation of the fibrotic stage was halted. This conclusion is reinforced by the decreased levels of PDGF-AB and Hyp, which are indicators of fibrotic changes.

To supplement these conclusions, we examined and compared the morphological changes of the four groups. The BLM/TED group exhibited no significant pathological changes in the alveolar cavities and no obvious morphological changes among the cell populations. The BLM group exhibited extensive pathological changes, with a reduction of alveolar space by 2 weeks and formation of obvious focal areas in the pulmonary tissue by 6 weeks. Additionally, examinations under high magnification revealed that fibroblasts occupied the main proportion of the pulmonary cell population by 6 weeks. In contrast, the alleviating effects of TED on inflammation and fibrosis were demonstrated in the BLM/TED group. By 6 weeks, no obvious changes occurred in the alveolar cavities and pulmonary mesenchyme.

## Conclusions

The co-administration of TED with BLM in the pulmonary tissues of mice significantly suppressed the inflammatory and fibrotic changes observed in the classic BLM models. The inflammatory response was suppressed from the initial stage (reduction of IL-8 levels), with no ‘bypassing’ effects to initiate the downstream process of inflammation. Additionally, the fibrotic process was also suppressed in terms of fibrotic marker quantification and morphological analysis, providing evidence to support our conclusion that TED is capable of suppressing the entire development process of BLM-induced pulmonary fibrosis in mice.
